# A Synopsis-Dental Ethics

**Published:** 1858-12

**Authors:** J. Taft


### A SYNOPSIS

Of some remarks made in a preliminary lecture to the Class in the Ohio College of Dental Surgery—Session 1858-9,

BY J. TAFT.

### DENTAL ETHICS.

The relation which the dentist and his patient sustain to each other, if not in theory, certainly in practice, is too often wholly overlooked by both parties. It is a relation in which there are duties and responsibilities devolving upon both.

When they assume that relation, each is indebted to the other, and they should both see to it, that the obligation is promptly honored. There are some things which the dentist has a right to expect from his patient; and the first we will mention is confidence—a *reliance* in his ability to do what he undertakes. I do not intend by this, that the patient should always believe that the operator has the ability to do every thing, but that it will be done it as well as by any one else, under the circumstances, and that the operations, when well cared for, and with favorable circumstances, will accomplish the design.

The patient should rely upon the judgement of the dentist who is to operate for him, and always yield to his opinion. It is the province of the dentist, to determine what is for the interest of his patient, at least so far as his teeth are concerned, and the duty of the patient to submit to the course suggested; if he does not, and from a want of confidence, he should not apply. It is proper that the patient should make suggestions, when it is presumed he has correct knowledge upon the subject; even then, however, the dentist should be the umpire.

The patient should have the most implicit confidence in the integrity of his dentist. The opportunity to practice imposition upon his patients, is constantly before him, and he who would take advantage of it, should not be trusted with the care of the teeth.

With the man, with any thing like correct principles, when he knows that his patient is relying implicitly upon him, in a matter so important as the welfare of his teeth, it is a stimulus to act uprightly; while suspicion would have the opposite tendency.

The patient should, at all times, be ready to appreciate good dental operations, and in speaking of them, give the dentist his just meed of praise; this is but justice to the operator. How often the opposite is done. We have oftentimes heard remarks, such as the following: "Oh yes, I have had my teeth filled, but I do not suppose it will do them any good."

Now, the patient, by such remarks, does the dentist great injustice, or does himself great injustice, by submitting to his operations; and when an operation has not fulfilled the expectation, great care should be exercised in speaking to others about it, unless the patient fully understands the subject in all its bearings. Much injustice is often done to the dentist in this respect. It is very seldom that a patient can understand all the circumstances that may influence an operation in his own mouth. Not one in a hundred is capable of doing this.

The following are some of the common circumstances that, to a greater or less extent, may affect the permanency, of fillings in the teeth. Teeth of imperfect structure, and easily acted upon by injurious agents; changes of the system, predisposing the teeth to decay; vitiation of the secretions of the mouth; the difficulty of operating, in some instances, owing to an unfavorable location of the cavity, thus preventing a definite view, and accurate manipulation.

The advanced stages of decay, and the difficulty of filling such teeth. The teeth are not always properly cared for, even after a good operation has been performed.

The operator is not always in a favorable mood for performing operations in a perfect manner. Every one is familiar with the fact, that improper temper, or nervous irritability, unfits for delicate and skillful operations. Yet the dentist is almost compelled to operate while in these conditions. Dental practice severely tries the patience and self-government of the operator.

It is the duty of the patient, always to co-operate with the dentist, for the permanency of his work, by keeping the teeth in good condition, so far as cleanliness and freedom from deposits is concerned.

No one has a right to complain of his dentist, if, after an operation, he has neglected his teeth. And no dentist who understands the obligations due him from his patient, would touch the teeth of such an one, without a very favorable prospect of reformation.

The patient should always be willing to pay the fee for a good operation.

No dentist will charge more for a good and efficient operation, than it is worth to the patient; indeed, no patient ever pays the full value of efficient operations; and the dentist usually knows better than any one, what his fee should be.

I refer now to *true men*, and not to those things, self-styled dentists,—whose operations, worse than worthless, are very dear at any price, indeed, costly without any fee at all. If the patient has the confidence in the moral honesty of his dentist he *should* have, he will not hesitate, for one moment, to honor his bills.

No one, who feels that his fees are low and grudgingly paid, will feel the same stimulus to improve and aim at a high point of excellence, as though it were otherwise. No operator can devote his time, labor, and material, to fill a tooth as well for one dollar, as for five; and yet the preservation of a tooth is worth vastly more, to any one, than this.

There are some obligations devolving upon the dentist to his patients.

The dentist should always manifest toward his patient, the bearing of a perfect gentleman. This is a suggestion applicable in all our intercourse with mankind, but, in a very high sense, it is true in regard to the dentist, whose contact with his patients is of so peculiar a character.

Kind attention should ever characterize the dentist; yet no unbecoming familiarity, nor sycophancy or fawning,—such a bearing is always disgusting to persons of a just appreciation of character.

Always exhibit sympathy for the suffering; yet permit not that sympathy to interfere with the prompt and efficient execution of an important operation; indeed, true sympathy stimulates to the most, through work.

True sympathy does not consist in honied words, and feeble, inefficient attempts to relieve from suffering,—but in prompt, energetic action, every stroke of which tells upon the work to be done.

The dentist should be prepared to fulfill the just expectations of his patients,—presuming the patient to be an enlightened one.

It is true, that persons, intelligent in other matters, but ignorant in regard to their teeth, oftentimes expect too much. It is the duty of the dentist, to correct such mistaken or false notions.

It is a very just expectation of the patient, when he applies to the dentist, that he will receive benefit or good at his hands,—either with reference to the preservation of the natural organs, or supplying the place of those lost, with artificial substitutes.

In no case should the dentist attempt to practice deception upon his patient. This, however, is frequently done, and the ignorance of the patient is given as an excuse. But we should remember, that any one, however ignorant he may be, who finds himself the subject of deception, will have rather a poor opinion of the moral uprightness of the operator, and will not be very likely to place himself in the power of such an one a second time.

But it is maintained, that it is better, oftentimes, to deceive children, in order to accomplish the desired end; indeed, we are often requested, by parents, to do this. It is better to employ force, if need be, to perform an operation for a child, than deception; for, it will invariably be found, that when the latter is once employed, for the next operation, force will be required; and if the child ever submits quietly to an operation, it is under the suspicion that a deception may, at any time, be practiced upon him; and the operator will find that it will be a long time, even with the most prudent action, before he can secure the confidence of his young patient.

The dentist should always perform his operations, in the best possible manner, under the circumstances. All operations upon the teeth can not be made alike perfect. The aim should be, to make every operation subserve a valuable purpose. I have noted, elsewhere in this paper, some of the circumstances that will modify the efficiency of an operation.

The manner has much to do with the success of the dentist. There are a great many little indescribable peculiarities of manner, that should not be overlooked, or disregarded. Those little amiabilities that we so much admire in all, should be especially cultivated by the dentist. These are things that will have their influence upon all classes of persons. Have much care for personal appearance. The attire should be clean, neat, and tasty, yet not flashy.

Cleanliness of person should be scrupulously regarded. The dentist who is slovenly and dirty, is so at his own cost. Some examples might be given here, but we leave the reader to supply his own illustrations.

Keep the hands perfectly clean, smooth, and soft; any one with rough, stiff hands, can not manipulate as delicately and skillfully, as though they were in good condition; with the latter condition, the sense of touch is far more perfect.

Much care is often necessary, to remove unpleasant odors from the hands, that may be contracted from a diseased mouth. No one likes to have dirty, offensive hands in the mouth, no matter how dirty it may be.

Keep the teeth clean, and in perfect condition. The advice and suggestions of the dentist to his patient, in regard to his teeth, come with rather a bad grace, through a set of dirty, filthy, unsightly, ragged teeth.

Keep a pure breath, as far as possible; and, in order to do this, use tobacco in no form, nor alcoholic spirits of any kind, nor any of those peculiar little luxuries that render the breath like a blast from a stygean pool. Common decency forbids that any one should blow the contaminated breath, from his own polluted carcass, into the mouth of those of natural tastes and delicate sensibilities. These things observe, and forget not.

Endeavor to make your patient as comfortable and as much at ease as possible, while in your presence. This is worthy of your attention.

We often hear remarks like the following: "I would much rather undergo the operation, than to endure the operator during the time." Of course, the patient would avoid such an operator, in future, if possible.

And now, gentlemen, if the hints and suggestions, given to you in this crude form, shall aid you, even in a slight degree, in forming your professional character, they will have accomplished their design.

